# Using Magnetically Responsive Tea Waste to Remove Lead in Waters under Environmentally Relevant Conditions

**DOI:** 10.1371/journal.pone.0066648

**Published:** 2013-06-20

**Authors:** Siang Yee Yeo, Siwon Choi, Vivian Dien, Yoke Keow Sow-Peh, Genggeng Qi, T. Alan Hatton, Patrick S. Doyle, Beng Joo Reginald Thio

**Affiliations:** 1 Engineering Product Development, Singapore University of Technology and Design, Singapore, Singapore; 2 Department of Chemical Engineering, Massachusetts Institute of Technology, Cambridge, Massachusetts, United States of America; 3 Department of Materials Science and Engineering, Massachusetts Institute of Technology, Cambridge, Massachusetts, United States of America; 4 Science Department, Hwa Chong Institution (High School), Singapore, Singapore; 5 Kaust-Cornell Center for Energy and Sustainability, Cornell University, Ithaca, New York, United States of America; National University of Singapore, Singapore

## Abstract

We report the use of a simple yet highly effective magnetite-waste tea composite to remove lead(II) (Pb^2+^) ions from water. Magnetite-waste tea composites were dispersed in four different types of water–deionized (DI), artificial rainwater, artificial groundwater and artificial freshwater–that mimic actual environmental conditions. The water samples had varying initial concentrations (0.16–5.55 ppm) of Pb^2+^ ions and were mixed with the magnetite-waste tea composite for at least 24 hours to allow adsorption of the Pb^2+^ ions to reach equilibrium. The magnetite-waste tea composites were stable in all the water samples for at least 3 months and could be easily removed from the aqueous media via the use of permanent magnets. We detected no significant leaching of iron (Fe) ions into the water from the magnetite-waste tea composites. The percentage of Pb adsorbed onto the magnetite-waste tea composite ranged from ∼70% to 100%; the composites were as effective as activated carbon (AC) in removing the Pb^2+^ ions from water, depending on the initial Pb concentration. Our prepared magnetite-waste tea composites show promise as a green, inexpensive and highly effective sorbent for removal of Pb in water under environmentally realistic conditions.

## Introduction

As industrialization continues worldwide, heavy metal contamination of the environment is becoming an increasing concern for its adverse health effects on human society. Heavy metals are released into the environment primarily via one of four main anthropogenic processes: the mining and smelting industries; manufacturing; agriculture; and fossil fuel burning power plants, vehicles, ships and aircrafts. Most heavy metals are toxic to humans. Specifically, lead (Pb) is a major cause of neurological diseases that impair basic mobility functions, and lead to growth defects in children [1]. Leakages of Pb ions into the water bodies generally occur as a result of corrosion of Pb-containing plumbing systems such as pipes and fittings, and surface run-offs of Pb-based materials (e.g. paints). Pb concentration levels in water systems around some industrial areas have been found to reach as high as 500 ppm [2], while the World Health Organization (WHO) recommends Pb concentrations below 0.01 ppm for safe drinking water [3].

The most common material used for heavy metal removal in contaminated waters is powdered activated carbon (AC) [4]. The high adsorption capacity of AC for the heavy metal ions can be attributed to its microporosity and high surface area to mass ratio [5]. However, the use of AC for water treatment can result in very high costs economically and environmentally. High temperatures of up to 800°C [6] are required to produce or “activate” the carbonaceous material, leading to high energy and capital costs [7]. This has provided motivation to search for a less expensive, more efficient natural adsorbent for removing heavy metals from wastewater.

Tea is very readily available in much of the world and has been proven to exhibit a high removal efficiency and sorption capacity for Pb^2+^ [8], [9]. The surface of tea leaves contains many polar, aliphatic and aromatic function groups [10] that allow it to successfully adsorb contaminants such as Pb^2+^, Ag^+^, Cu^2+^ and Al^3+^ ions [8]. Waste tea from local restaurants and cafes could be utilized to create a secondary use for a product that is already widely consumed in our societies. Magnetization of the tea waste (via the synthesis of a magnetite or Fe_3_O_4_ coating) can also serve as a more efficient, easier and faster way of removing the adsorbent-contaminant complex in place of filtration or flocculation. However, iron (Fe) can serve as a micronutrient that promotes algal bloom in inland and coastal waters [11]. Hence it is critical that the introduction of magnetite-waste tea composites as sorbents for Pb removal in contaminated waters do not accidentally create algal blooms with the release of large amounts of Fe ions into the waters.

The objective of this study is to quantify the Pb^2+^ sorption capacity of magnetically responsive waste tea (magnetite-waste tea composite) under environmental conditions similar to those found in natural waters which are typically used for drinking needs. These include but are not limited to rainwater, groundwater and freshwater. Numerous studies [8], [12], [13], [14] have been published that demonstrated the effectiveness of tea leaves as sorbents for Pb^2+^. However, their studies were performed in acidic media with varying initial Pb concentrations of up to 400 ppm and in the absence of natural organic matter (NOM); such conditions are not representative of actual real-world conditions. Further, [Pb^2+^] at concentrations above 100 ppm have been reported to precipitate at pH >5.3 [8], making pH effect sorption studies difficult if not impossible. For these reasons in this work, we varied the initial [Pb^2+^] from 0.1–5 ppm in artificial waters to allow us to understand the Pb sorption capacity and performance of the magnetite-waste tea composite in the simulated environmentally-relevant conditions.

## Materials and Methods

### Materials

Tea waste (Lipton Red Tea and Dilmah Ceylon Black Tea) were obtained from the Hwa Chong Institution (HCI) and Singapore University of Technology and Design (SUTD) cafeterias, washed with hot boiling distilled water 3 times and then rinsed with deionized (DI) water 7 times to remove any traces of impurities. The tea dusts were then dried overnight in an oven at 70°C, and subsequently stored in plastic vials in dessicators for use as sorbents in the sorption experiments.

Iron(II) sulfate (Chemicon), iron(III) chloride (Scharlau), hydrochloric acid (Honeywell), ethanol (Merck, Germany), Darco G-60 100 mesh activated carbon, AC (Sigma-Aldrich) and aqueous ammonia (Honeywell) were all analytical grade reagents and used as-is without further purification. The stock standard solution of 1000 ppm Pb(NO_3_)_2_ was of atomic absorption spectroscopy (AAS grade) and purchased from Perkin Elmer (Waltham, MA).

### Synthesis and Characterization of Magnetite-waste Tea Composite

The synthesis of the magnetite-waste tea composite was performed using the method adapted from a previous study involving the preparation of magnetic ragweed pollen grains [15]. First, 13.31 g of FeCl_3_•6 H_2_O, 27.77 g of FeSO_4_•7 H_2_O, 45 ml of DI water, 5 ml of 5 M HCl and 5 ml of ethanol were mixed in a 100 ml beaker, followed by stirring and heating to 90°C, to make sure all Fe salts were fully dissolved. Second, 1 g of tea waste powder was dispersed in 30 ml of the Fe solution and stirred for 48 hours at room temperature on a magnetic stirrer to allow for the complexation of tea with the Fe salts. After the 2 days, the remaining iron solution was filtered off using a vacuum pump and the tea was rinsed briefly with DI water. Third, the filtered tea was then transferred into a clean beaker and 20 ml of 25% (w/v) aqueous ammonia was added. After 2 hours, the magnetite-waste tea was again filtered to remove the ammonia and rinsed with DI water. Finally, the magnetite-waste tea composite was placed on glass petri dishes and left to dry overnight in the oven at 60°C, and then subsequently stored in plastic vials. The synthesis steps for magnetite are similar to the magnetite-waste tea composites except that the tea powders were not added.

The morphologies of both uncoated tea waste and magnetite-waste tea were obtained using a scanning electron microscope (SEM JEOL-6060SEM, JEOL Ltd, Japan). All samples for SEM were sputter-coated with platinum prior to imaging.

Vibrating Sample Magnetometry (VSM Model 1660, ADE) was used to obtain the magnetization (*M-H*) curve of magnetite-waste tea composite powders at room temperature. Each sample was prepared by fixing 20–80 mg of the powder onto a glass coverslip and fixing the coverslip to the VSM sample holder. All parts were held together by double-sided adhesive tape.

Thermogravimetric Analyses (TGA) of both uncoated and magnetite-waste tea composite powders were performed on a TGA Q50 (TA Instruments). 5 to 15 mg of each sample was taken for measurement under a constant nitrogen flow rate of 100 mL/min. The temperature was raised from 25°C to 980°C at a rate of 20°C/min.

Nitrogen physisorption isotherms were measured at 77 K using a Micromeritics ASAP 2020 analyzer. The samples (uncoated tea and magnetite-waste tea composite powders) were degassed at 323 K under vacuum for 24 hours prior to the measurements. The specific surface areas of the samples were calculated by the Brunauer-Emmett-Teller (BET) method [16]. The pore sizes of the samples were calculated using the Barrett–Joyner–Halenda (BJH) model [17].

### Preparation of Artificial Waters

DI water (Mili-Q, Millipore) with a resistivity of 18.2 MΩ.cm was used to prepare the artificial waters, with the appropriate amounts of key electrolytes and dissolved organic contents [18], [19], [20]. All three of the artificial waters (rainwater, groundwater and freshwater) were prepared by dissolving similar amounts of the salts (NaCl, CaCl_2_, MgCl_2_ and NaHCO_3_) commonly found in real natural waters while organic content was represented using humic acid (Sigma-Aldrich). About half the given mass of humic acid is made up of carbon [21]. The concentrations of the key electrolytes and organic carbon in the various artificial waters are given in Table 1.

**Table 1:** Concentrations of main electrolytes and dissolved organic content in the artificial waters used in the experiments.

	Units	Artificial Freshwater	Artificial Groundwater	Artificial Rainwater	DI Water
pH		8.4	7.1	4.2	6.3
TOC	µM C	5200	1564	–	–
Na^+^	mg/L	50	45	19.4	–
Ca^2+^	mg/L	26.5	87	22.4	–
Cl^−^	mg/L	124.6	155	60.7	–
HCO_3_^−^	mg/L	–	120	–	–

### Fe Leaching and Magnetite-waste Tea Composite Stability Tests

Iron (Fe) has been shown in studies to be a critical nutrient in promoting harmful algal blooms [11], [22]. In order to determine the stability of the magnetite coating on the tea, leaching tests were performed to observe the amount of Fe that would leach out of the magnetite-waste tea composite in all 4 water types. 50 mg of magnetite-waste tea composite was dispersed in 10 ml of water sample in a 15 ml centrifuge tube (BD Falcon), and left on a shaker for 3 consecutive days. 3 samples (triplicates) were removed after 24 hours (Day 1), 48 hours (Day 2), and 72 hours (Day 3) respectively, for each water type. The samples were then centrifuged at 8000 rpm for 5 minutes. A calibration curve of Fe standards (1, 2, 5 ppm) was run on the Flame-Atomic Absorption Spectrometer (AAS, AA-6300 Shimadzu), and the water samples were analyzed on the AAS for iron content.

### Batch Adsorption Tests

Batch sorption experiments to evaluate the efficiency of removal of Pb^2+^ ions by the tea wastes (both uncoated and magnetite coated), magnetite and AC were carried out in 15 mL centrifuge tubes (BD Falcon) at room temperature. 30 mg of each sorbent was added to 10 mL of water with varying initial Pb^2+^ concentrations. Starting Pb^2+^ concentrations ranged from 0.16–5.55 ppm; these values are representative of the typical Pb^2+^ concentrations in environmentally realistic Pb–contaminated waters [23], [24]. Samples were placed on an orbital shaker (Stuart) for 24 hours to allow the adsorption to achieve equilibrium and to fully disperse the adsorbents in the artificial waters. The samples were then centrifuged (Beckman Coulter) at 8000 rpm for 5 minutes prior to sampling by an atomic absorption spectrophotometer (AAS, Shimadzu AA-6300) for lead content. A calibration curve of Pb standards (0.1, 1, 2 and 5 ppm) was run on the AAS with each batch of sorption experiments. All batch adsorption tests were carried out at least thrice. One-way analysis of variance (ANOVA) at 95% confidence level (Microsoft Excel 2010) was used to check for statistically significant differences in the batch Pb^2+^ sorption experiments between the four different sorbents under the same solution conditions. Differences in Pb^2+^ sorption results with *p*-value <0.05 are considered to be statistically significant.

### Zeta Potential Measurements

The electrophoretic mobility (EPM) of the various sorbents (tea wastes, magnetite and AC) was characterized using Laser Doppler velocimetry (Malvern Zetasizer Nano ZS-90), with the EPMs converted to ζ-potentials using the Smoluchowski equation [25]. Before each measurement, the sample was subjected to ultrasonic treatment for several minutes to disperse the sorbents in suspension thoroughly. 5 measurements were conducted for each sample.

## Results and Discussion

### Coating and Characterization of Magnetite onto Tea Waste

**Figure 1:**
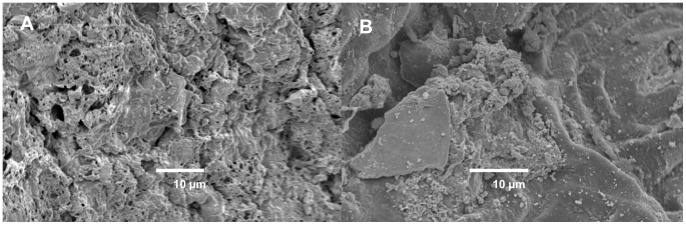
SEM images of waste tea powder (A) without and (B) with magnetite coating.

The magnetite-waste tea composite was successfully synthesized by using the co-precipitation of Fe(II) and Fe(III) salts in aqueous ammonia and is shown in Figure 1. The BET surface areas for the uncoated tea, magnetite-waste tea composite and activated carbon (AC) were measured to be 1.04, 6.00 and 3350 m^2^/g respectively, while the pore sizes were calculated to be 160, 125 and 328 Å. Table 2 summarizes the key parameters of the sorbents measured using the nitrogen physisorption isotherms.

**Table 2:** Key parameters of the tea powders (unmagnetized tea and magnetite-waste tea composite) measured using the nitrogen physisorption isotherms.

	Units	Unmagnetized tea	Magnetite-waste tea composite	AC
BET surface area	m^2^/g	1.04	6.00	3350
Pore size	Å	160	125	32

**Figure 2:**
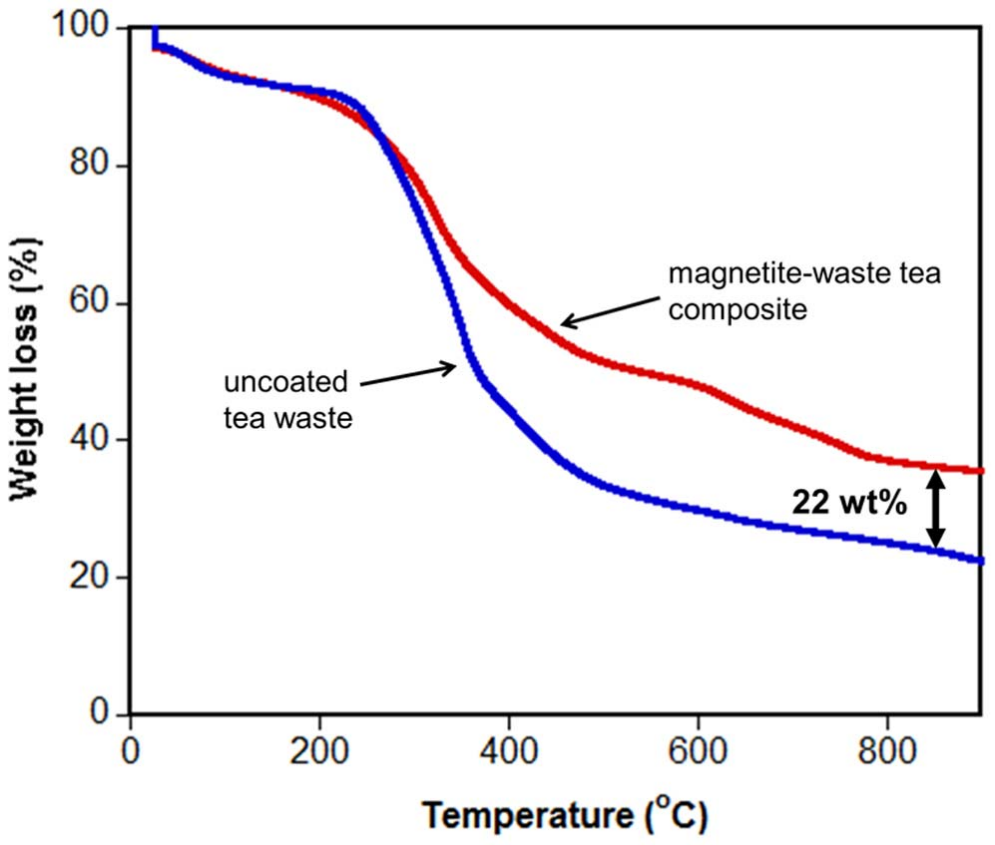
TGA analyses of the uncoated and magnetite-waste tea composite showing that the magnetite coating accounts for about 22% of the total mass of the composite.

Thermogravimetric analysis (TGA) of the uncoated and magnetite-waste tea composite (Figure 2) showed that the magnetite coating contributed about 22 wt% to the total mass of the magnetite-waste tea composite.

### Magnetic Property of the Magnetite-waste Tea Composite

**Figure 3:**
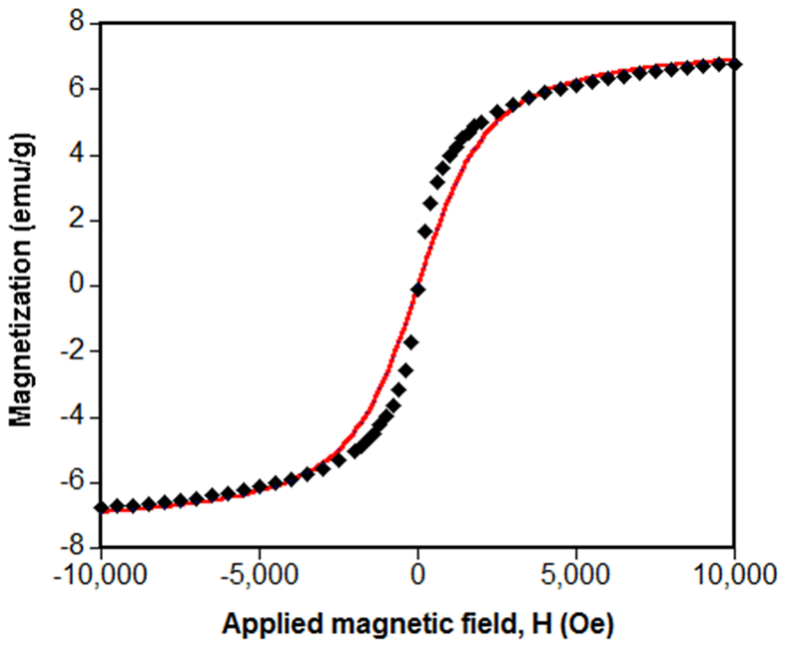
Magnetization curve of the magnetite-waste tea composite at room temperature. The points were obtained from VSM measurement, while the solid line was the best fit from the Langevin function assuming the composite particles were monodisperse.

The magnetic behavior of the magnetite-waste tea composite was analyzed by VSM at 298 K and the externally applied magnetic field was cycled between -10 and 10 kOe. Figure 3 shows that the magnetite-waste tea composite is superparamagnetic, given the absence of a hysteresis loop. The saturation magnetization (*M_s_*) of the magnetite-waste tea composite is 7 emu/g. Since magnetite accounts for about 22 wt% of the total mass of the composite, the *M_s_* value of the magnetite without the tea waste can be calculated as 31.8 emu/g. Bulk Fe_3_O_4_ has a *M_s_* value of 92 emu/g [26]. The smaller *M_s_* value of the magnetite in the waste tea complex compared with the bulk Fe_3_O_4_ value can be attributed to the reduced primary grain size of the magnetite coating on the tea [26] and the interaction of organics from the tea with the iron oxide, both of which decrease *M_s_* [27].

The magnetization *M* of the magnetite-waste tea composite assuming a monodisperse particle diameter d in the direction of an applied magnetic field H can be modeled using the Langevin function *L(α).*

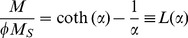
(1)where *φ* is the solid volume fraction, *M_s_* is the saturation magnetization of the bulk magnetite, *α = πμ_0_M_s_Hd^3^/6kT*, *μ_0_* is the vacuum permeability, *k* is the Boltzmann constant and *T* is the temperature [28].

From this equation, the solid volume fraction of magnetite in the magnetite-waste tea composite can be obtained by fitting the Langevin function to the experimental magnetization curve. The best fit solid line in Figure 3 was obtained when *φ* = 0.08. This indicates that while magnetite makes up 22% by mass of the composite it occupies only 8% of the solid’s volume. Tea waste is less dense than magnetite.

**Figure 4:**
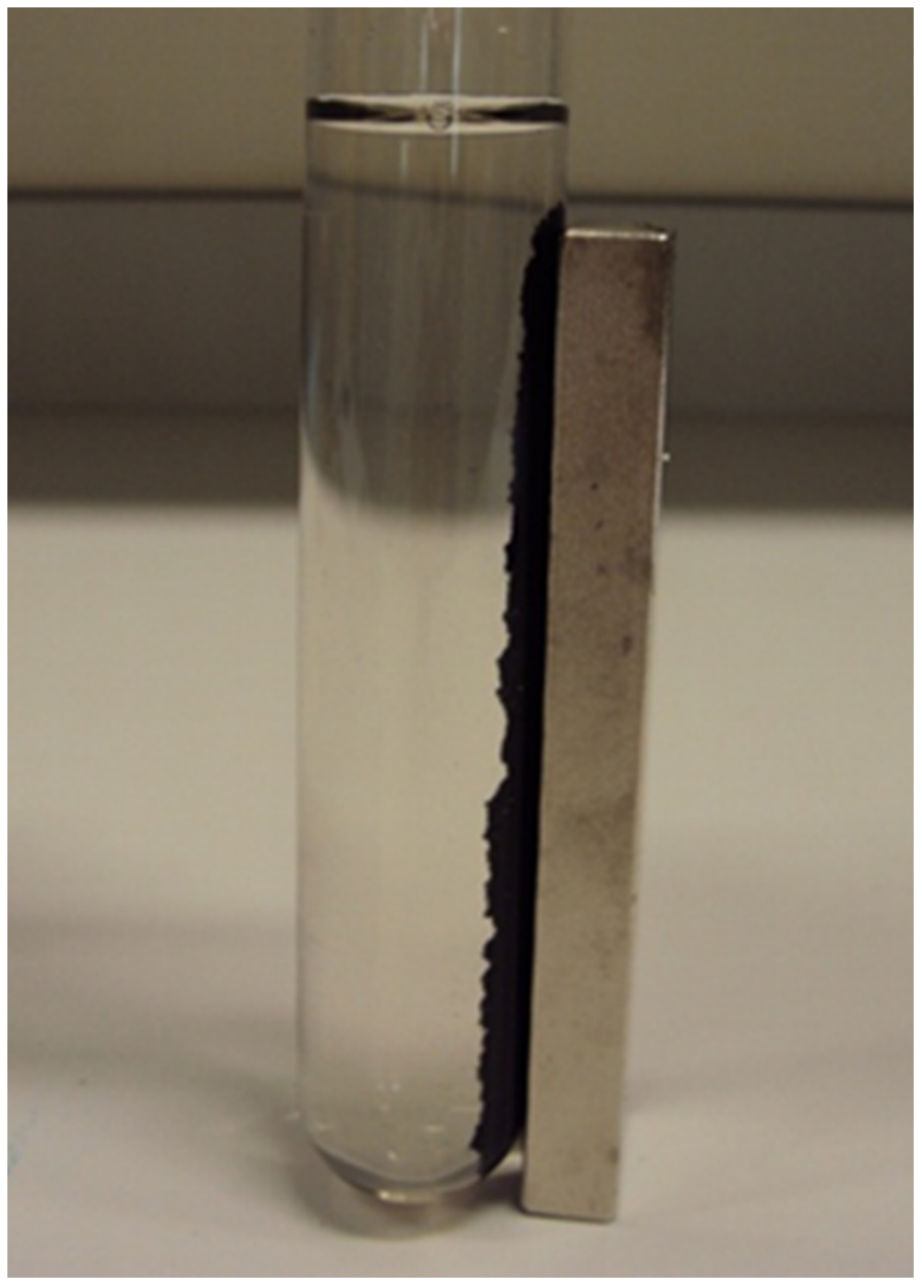
Testing the magnetic stability of magnetite-waste tea composite after 8 days in water.

Figure 4 illustrates the easy separation of the magnetite-waste tea composite from water via the use of a rare earth permanent magnet. The composite is stable in the artificial waters and remains attracted to a magnet for up to at least 3 months.

### Fe Leaching from the Magnetite-waste Tea Composite into the Artificial Waters

**Figure 5:**
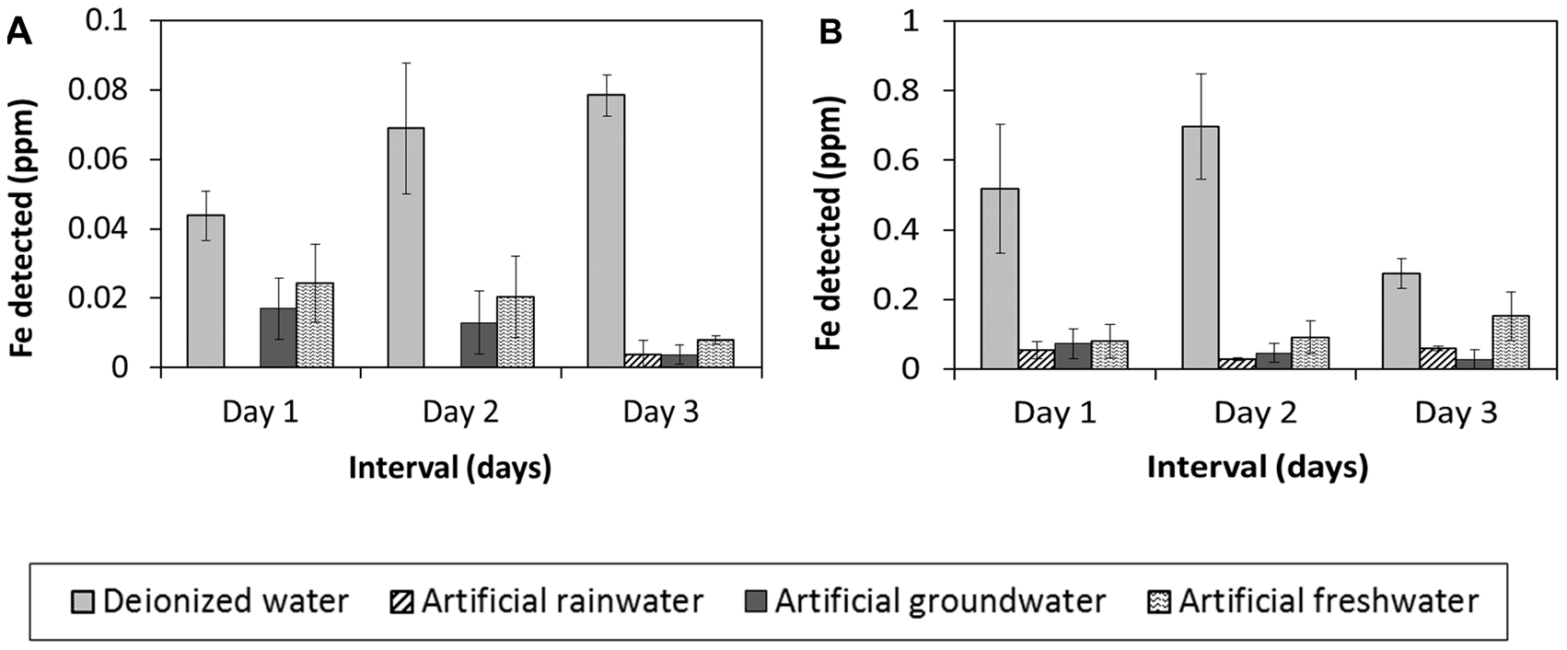
Amount of Fe leached out into the four waters from uncoated tea (A) and magnetite-waste tea composite (B).

Figure 5 shows that uncoated tea leached low concentrations of Fe in all 4 types of waters (≤0.08 ppm) while the magnetite-waste tea composite leached Fe concentrations of ≤0.7 ppm. The magnetite-waste tea composite has more Fe leaching due to the magnetite layer coated onto the waste tea dusts during the magnetization procedure. According to WHO guidelines [29], approximately 0.5–50 ppm of Fe can be found naturally in fresh waters. Our data show that the magnetite coating of tea does not cause significant leaching of Fe into the waters in concentrations sufficiently high that it will pose a health hazard. The concentrations of Fe leaching from both uncoated tea and magnetite-waste tea were similar to Fe concentrations found in the natural waters.

### Choice of Solution pH

In this study, the pH values of the artificial waters were adjusted to be 4.2 for rainwater, 7.1 for groundwater and 8.4 for freshwater while the [Pb^2+^] was varied from 0.15–5.5 ppm. The pH value of DI water was measured to be 6.3. From previous studies of Pb sorption onto tea [8], [14], the optimum pH fell in the range of 4–6. Both Liu et al. and Amarasinghe et al. observed in their studies that for [Pb^2+^]>100 ppm and at pH >5.3, Pb^2+^ ions begin to hydrolyze and precipitate out, making it impossible to conduct sorption studies when pH exceeds 6. In addition, the adsorption of metal ions at low pH is known to be poor as there would be competition with the H^+^ ions for binding to the active sites on the tea surface. Their studies were conducted in solution pH 4–5.5 with [Pb^2+^] varying from 0–400 ppm. However, the Pb sorption onto tea under such conditions is not environmentally realistic. Ambient waters have pH values between 7–8, with the exception of rainwater which is typically acidic and has a pH ∼4 [20]. Typical [Pb^2+^] in highly contaminated waters range from 0.1 to 0.7 ppm [23], [24]. The WHO health based guideline limit for maximum Pb in drinking water is 0.01 ppm [3]. This work thus investigates the potential of using magnetized tea waste under environmentally relevant operating conditions to remove Pb^2+^ ions from aqueous solutions.

### Effect of Sorbent Type

**Figure 6:**
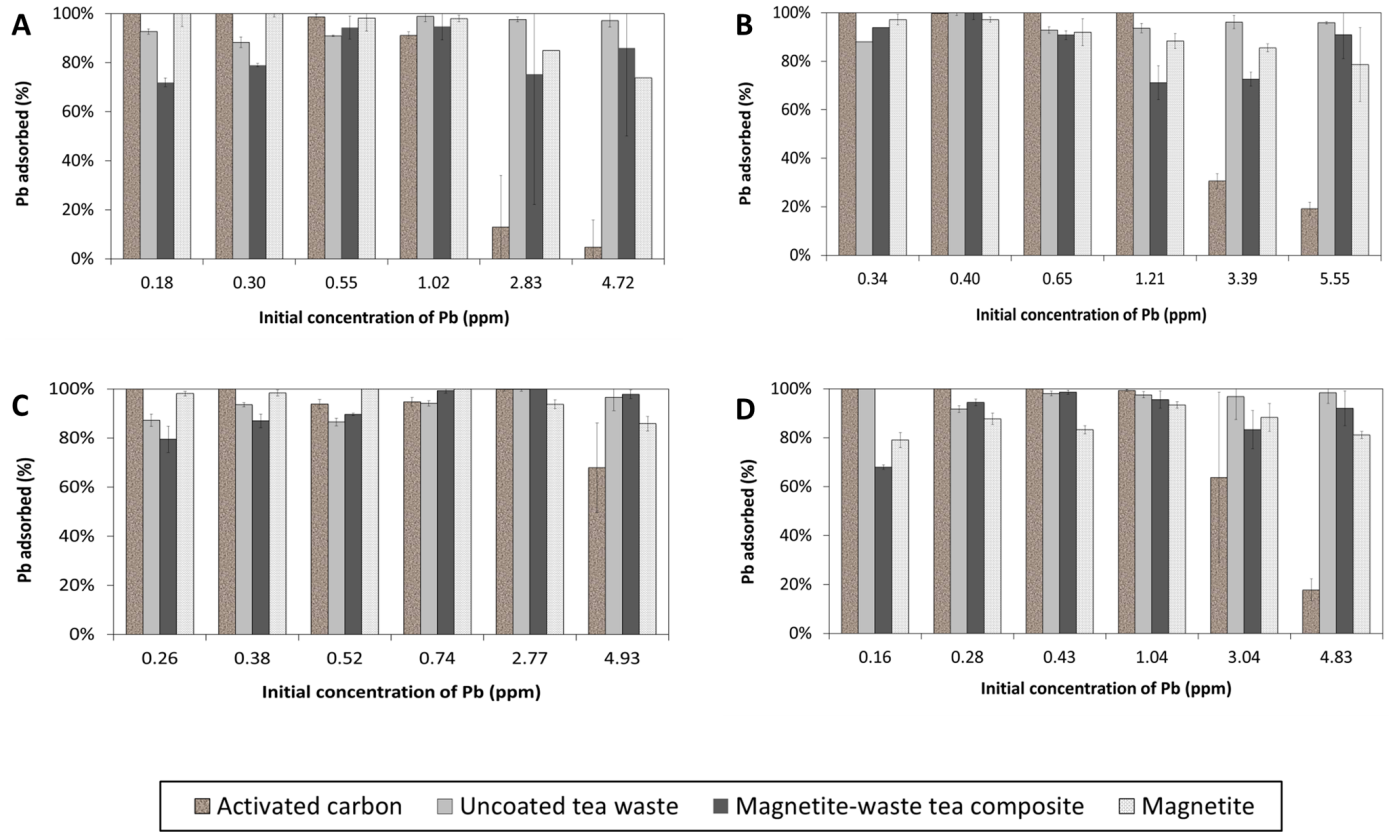
Percentage of Pb adsorbed onto the four sorbents for each of the four different waters: (A) deionized water, (B) rainwater, (C) groundwater and (D) freshwater.

AC is the most commonly used sorbent for removal of both inorganic and organic contaminants in polluted waters. Experiments were conducted to compare the Pb^2+^ sorption performance of AC with those of the uncoated tea and magnetite-waste tea composites. Magnetite’s capability as a sorbent was also tested. The results are presented in Figure 6. AC shows a reduced Pb^2+^ percentage removal in all 4 waters compared to magnetite and both uncoated and magnetite-waste tea when the initial Pb concentrations were above 2 ppm. AC has a larger surface area compared to both the uncoated and magnetite-waste tea. The observed reduction in Pb sorption by AC could be partly due to the agglomeration of Pb^2+^ ions covering the pores on the surface of AC, thus blocking free Pb^2+^ ions in the solution from binding to the AC surface.

The removal/recovery of simple Pb–tea sorbents from the treated waters poses a challenge. Magnetization of the sorbents (via the coating of magnetite) allows a quick and easy removal/recovery of used sorbents via the simple application of an external magnetic field.

Among the 4 sorbents tested, uncoated tea has the highest sorption for Pb^2+^ by adsorbing at least 86% of a range of varying initial [Pb^2+^] for all waters. While the uncoated tea powder does not have a very porous structure to provide a high surface area for adsorption of Pb^2+^ (1.04 m^2^/g versus 3350 m^2^/g for AC), tea dusts/leaves are known to contain numerous functional groups [10], of which the amine group (−NH_2_), aromatic and aliphatic carbons, and carboxylic carbons (−COOH) play an active role in sorption of Pb [8]. Our results (Figure 6) indicate that both the uncoated tea and magnetite-waste tea composite have significant differences (*p*<0.05) in Pb sorption performance compared to AC in twenty out of twenty-four of the solution conditions. These differences can be partially attributed to the smaller BET surface areas on the teas compared to AC, limiting the number of available sorption sites for Pb^2+^ to bind, in addition to the variations in binding affinity of the sorbents for Pb^2+^ between AC, uncoated tea and magnetite-waste tea composite. In all four water types, both the uncoated tea and magnetite-waste tea composite adsorbed more Pb^2+^ than AC when the initial [Pb^2+^] exceeds 2 ppm while AC is a more effective sorbent when initial [Pb^2+^] is less than 2 ppm. The uncoated waste tea’s Pb^2+^ sorption capacity is similar to that of other studies [8], [10], [14] which show that tea wastes can be a potential low cost alternative to other adsorbents such as AC. From Figure 6, the results for Pb^2+^ sorption by the magnetite-waste tea composite samples are comparable to that of uncoated tea for most cases, with the exception of a decrease of ∼30% in Pb^2+^ sorption for freshwater with an initial [Pb^2+^] of 0.16 ppm.

### Effect of Water Type

**Figure 7:**
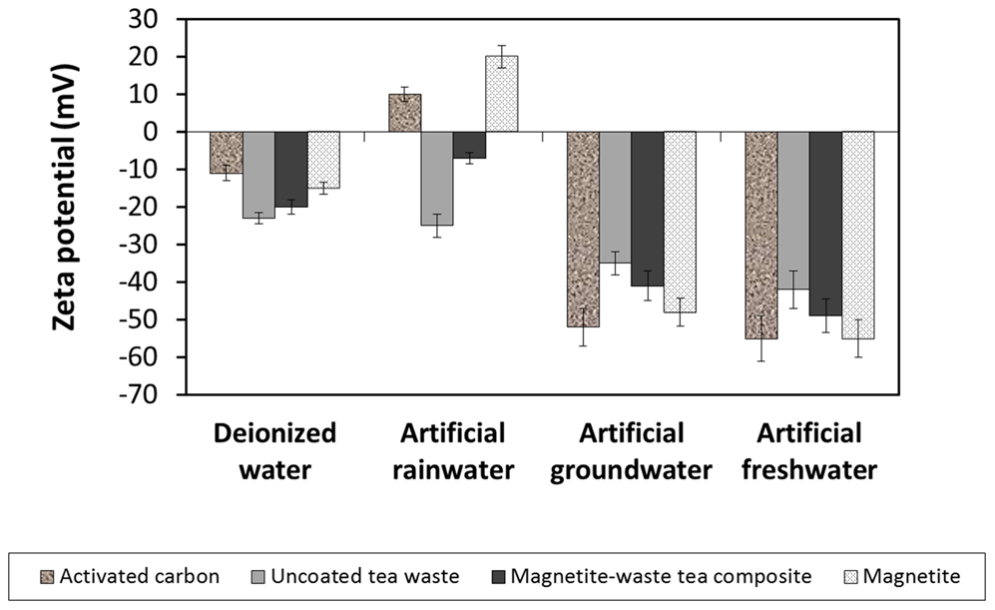
ζ-potentials of the various sorbents dispersed in the artificial waters.

The percentage of Pb removal by the magnetite-waste tea composite in all the four waters was at least 70%. The sorption of Pb by the composite was higher in both artificial groundwater and artificial freshwater (>80%) compared to DI and artificial rainwater (>70%). The humic acid content found in freshwater and groundwater (Table 1) could be a main contributing factor to this increase in Pb sorption and removal. Numerous studies have reported that humic acid acts as a chelating agent and forms complexes with metal ions [30], [31], [32]. Humic acid contains phenolic hydroxyl (−OH) and carboxylic groups (−COOH) which are critical for the formation of the Pb-humate complex [33], [34]. The formation of these Pb-humate complexes could have aided the increase of Pb sorption onto the surface of magnetite-waste tea complexes. Furthermore the presence of Ca^2+^ ions in groundwater and freshwater can act as a bridge [21], [35] between the negatively charged Pb-humate complex and the similarly charged magnetite-waste tea composite (Figure 7), enhancing the adsorption of Pb.

Pb sorption for uncoated tea waste ranged from 86–100% while Pb sorption for magnetite ranged from 73–100%. Pb sorption by AC displayed the same trend, with 100% sorption for initial [Pb^2+^] <1.5 ppm, and a decrease in Pb adsorption at initial concentrations of 3–5 ppm for all waters. The presence or absence of humic acids did not affect the Pb sorption by the uncoated tea waste, AC or magnetite. Figure 7 shows that all four types of sorbents were negatively charged in water with some exceptions in artificial rainwater. This suggests that electrostatic charge and humic acid are insufficient in explaining the increase of Pb adsorption by the magnetite-waste tea composite, and that enhanced Pb sorption requires the presence of humic substances and both the iron oxide and organics on the tea surface. In many systems involving natural organic matter (NOM), ζ-potential does not adequately predict adsorption. Other mechanisms such as steric interactions can enhance or impede adsorption even under electrostatically repulsive or attractive conditions [36].

### Conclusions

This study shows the effective use of magnetite-waste tea as an inexpensive and green sorbent to remove Pb in contaminated waters under environmentally relevant conditions. Our data from the Fe leaching tests indicate that both uncoated tea and magnetite-waste tea composite release very low concentrations of Fe into the waters (<0.08 and 0.7 ppm respectively), and at levels that do not exceed the Fe concentrations found naturally in the environment. The magnetic attraction tests demonstrated that the magnetite-waste tea composite remains stable and retains its magnetic properties while being dispersed in the simulated natural waters for at least 3 months. This allows for easy separation of the Pb-laden sorbents from treated waters. The magnetite-waste tea composite is a better material than AC in removing Pb from waters with initial Pb concentrations >2 ppm. The presence of humic acid in artificial freshwater and groundwater enhances the sorption of Pb by the magnetite-waste tea composite. Given the availability of tea wastes worldwide, magnetically responsive tea wastes have considerable potential to be used as a low cost sorbent in treating Pb bearing waters. The mass of iron oxide coating on the magnetite-waste tea composite can be optimized to reduce cost and wastage of the Fe salts without sacrificing its magnetic performance. Finally, both the Fe and Pb can be recovered by incineration of the magnetite-waste tea composite.
